# Examining the impact of validated handover protocols on treatment outcomes in polytrauma patients: a systematic review

**DOI:** 10.1007/s00068-025-02776-z

**Published:** 2025-02-18

**Authors:** Eva Steinfeld, Karolina Dahms, Julia Dormann, Kelly Ansems, Heidrun Janka, Maria Inti-Metzendorf, Gernot Marx, Carina Benstoem, Thomas Breuer

**Affiliations:** 1 Department of Intensive Care Medicine and Intermediate Care, Medical Faculty, RWTH Aachen University, Aachen, Germany; 2 Institute of General Practice, Medical Faculty of the Heinrich-Heine-University Dusseldorf, Dusseldorf, Germany

**Keywords:** Handover, Patient handoff, Polytrauma, Intensive care

## Abstract

**Purpose:**

Effective patient handovers in healthcare settings are critical for ensuring patient safety and care quality. Handover tools have gained prominence as potential aids in improving these transitions. This systematic review seeks to answer the question if the use of validated handover protocols leads to better treatment outcomes in polytrauma patients compared to no use of validated handover protocols.

**Methods:**

We searched PubMed, Cochrane Central Register of Controlled Trials and Web of Science to identify relevant studies from inception of each database to June 15, 2022. We intended to include systematic reviews and randomized controlled trials comparing the use of validated handover tools to no use of such tools in adult polytrauma patients.

**Results:**

Despite the absence of systematic reviews and RCTs meeting our criteria, we included 26 initially excluded studies to glean insights into handover tool usage. This broader inclusion facilitated the identification of two categories of tools: standardized tools and customized tools. Among studies employing customized tools, positive outcomes were reported in various aspects, including enhanced information quality, improved staff communication, and reduced risks and treatment errors. In contrast, studies utilizing well-established standardized tools documented improvements in communication, documentation, and overall satisfaction among medical professionals, signaling a reduction in communication errors and lost information.

**Conclusion:**

Heterogeneity of the studies and no trials meeting our eligibility criteria present challenges for conducting a traditional systematic review. In the lack of evidence from RCTs and systematic reviews, our analysis of the available studies sheds light on the complexities of assessing handover tools’ utility, especially in diverse clinical settings. It highlights the need for more standardized methodologies and further investigation into the effectiveness of custom-designed tools. It emphasizes the importance of understanding the role of handover tools in healthcare. While some studies suggest positive outcomes, further research is necessary to elucidate the design and implementation of these tools to enhance care and support healthcare professionals in their roles.

## Introduction

The process of transferring patients to the Intensive Care Unit (ICU) encompasses a crucial aspect: the handover of comprehensive patient data. The integrity of this data automatically influences the success of the subsequent patient care [1]. An imperfect or incomplete transfer process and communication can potentially lead to complications in patient care or even massive treatment errors [1]. An analysis of events has shown that 67% of “fatal errors” can be traced back to communication error [2]. In this review, we focused on polytrauma patients in the ICU, individuals who have sustained multiple, and life-threatening injuries. Their care demands the coordinated efforts of multidisciplinary healthcare teams and increases the complexity of information transmission during handovers [3].

In order to enable these handovers to be structured and complete, tools are used. These tools encompass standardized protocols, guidelines, and communication frameworks, and their adoption has been spurred by the quest to improve patient care, minimize errors, and enhance clinical outcomes.

Different studies have already explored the consequences of poor handovers for patients and what content should be communicated and thus included in standardized tools [4, 5].

In this study, we focus on the effect of handover tools for the particularly vulnerable group of polytrauma patients. Due to the diversity of handovers in the context of polytrauma care, the objective of this systematic review is to answer the following research question: Does the use of validated handover protocols lead to better treatment outcomes in polytrauma patients compared to no use of validated handover protocols?

### Methods

This review is part of the guideline project ‘S3-Leitlinie Intensivmedizin nach Polytrauma’ (AWMF Nr. 040-014) guided by the German Interdisciplinary Association of Critical Care and Emergency Medicine (Deutsche Interdisziplinäre Vereinigung für Intensiv- und Notfallmedizin, DIVI) and the German Society for Anaesthesiology and Intensive Care Medicine (Deutsche Gesellschaft für Anästhesiologie und Intensivmedizin, DGAI). The aim was to summarize the current evidence in the field of polytrauma to formulate specific recommendations. All studies that were carried out as part of this project used the same methodology which was consented within the guideline group.

### Eligibility criteria

We included studies comparing the use of validated handover tools with no use of validated handover tools in adult polytrauma patients admitted to the ICU that met the following inclusion criteria:

- Age of the included patients is ≥ 18 years
- Polytrauma present and defined as: a simultaneous injury to multiple body regions or organ systems, at least one or more of which, in combination, is life-threatening [5]
- Randomized controlled trial (RCT) or systematic review that includes RCTs
- Language of publication: English or German
- No multiple publication without additional information
- Publication accessible as full text
- Comparison of use of validated handover tools with no use

### Search strategy

We conducted a systematic search in PubMed, Cochrane Central Register of Controlled Trials (CENTRAL) and Web of Science (Science Citation Index Expanded und Emerging Sources Citation Index) from inception of each database to June 15, 2022 with no restrictions on the language of publication. Details of our search strategy are provided in the Appendix No 1. In addition, we searched reference lists of included studies to identify other potentially eligible studies.

#### Study selection

We imported the records from the systematic search into the Rayyan Systematic Review App (Rayyan, Cambridge, MA, USA). Three authors independently screened the titles and abstracts of all potential studies. Included full-text study publications were retrieved, imported into Microsoft Excel (Microsoft, Redmond, WA, USA) and screened by two authors independently. Reasons for exclusion of ineligible studies were recorded. Any disagreements were resolved through discussion or, if required, consultation with a third author.

### Data collection process

One reviewer extracted study and outcome data into a customized data collection form developed in Microsoft Excel, which was checked by a second investigator. Any disagreements were resolved by discussion or by consulting a third review author, if necessary.

We extracted the following outcome measures. To avoid selective reporting bias, we included studies regardless of the reported outcome data.

The following data were obtained:

1. Study characteristics: authors, publication date, and study design
2. Participants characteristics: number of included participants, gender, age
3. Intervention: handover tool, comparator, from station A to station B
4. Clinical outcomes: all-cause mortality (day 28, day 60, time to-event, and up to longest follow-up), clinical status (duration of mechanical ventilation, need for mechanical ventilation), serious adverse events (SAE), adverse events (AE), infections, quality of life.

We transmitted the outcome data into a statistical software (RevMan 5.3, Cochrane, London, England). Missing data resulted in the exclusion of the study in the analyses of the missing outcome.

### Study risk of bias assessment

The risk of bias of included studies was assessed by two authors independently using the Risk of Bias 2 (RoB 2) tool (Cochrane, London, England). This tool addresses five domains of bias (randomization process, deviations from intended interventions, missing outcome data, measurement of the outcome, selection of the reported results). The signaling questions of the tool were used to make a judgement according to the available options. We used the algorithms proposed in RoB 2 to assign each domain and the overall risk of bias to a level of bias: low risk of bias, some concerns, high risk of bias. Any disagreements between reviewers were resolved by discussion or by involvement of another author.

### Measures of treatment effect

No continuous or dichotomous outcomes were extracted.

### Data synthesis

We would have performed meta-analyses only, if the clinical and methodological characteristics of individual studies were sufficiently homogeneous.

#### Narrative presentation

The included studies did not report predefined endpoints as originally planned. However, the diverse studies offered an opportunity to assess whether handover tools had a favorable impact, either on patient care in a broad sense or on the well-being of hospital staff.

## Results

### Study selection

**Fig. 1:**
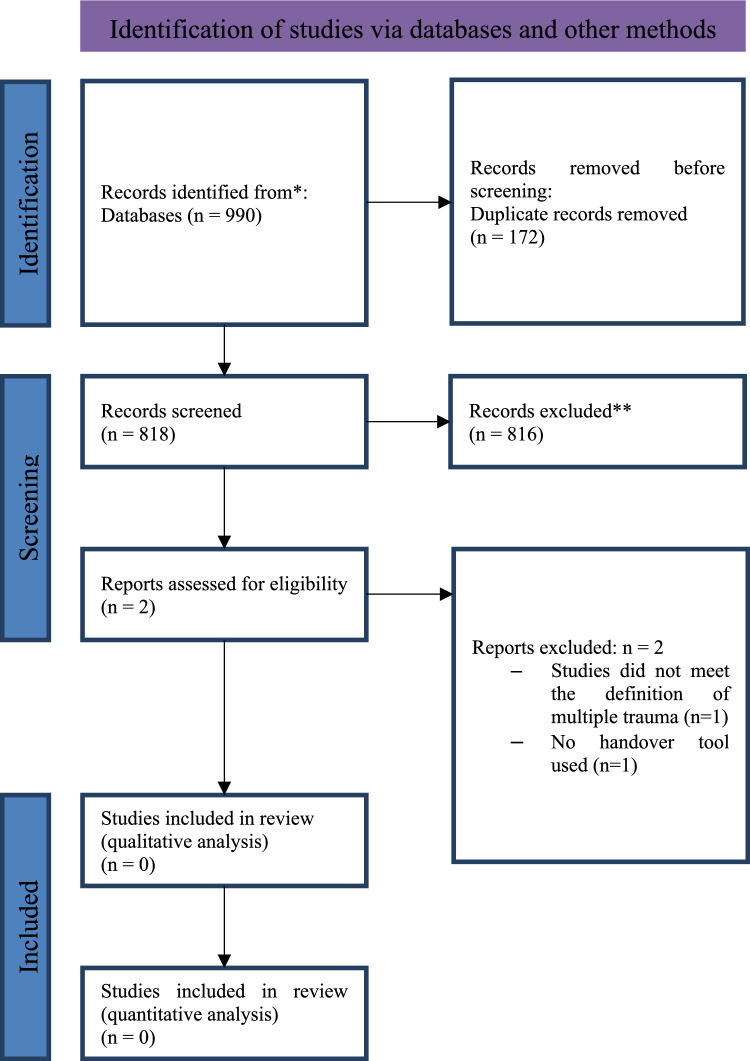
Flow chart of the systematic review selection process

The search identified 990 records (Fig. 1). After removing all duplicates, title abstract screening was performed on 818 records. After that, 815 studies were excluded and of the remaining two studies the full texts were screened. Reasons for exclusion of these two studies were “not meeting the definition of multiple trauma” (n = 1 [6];) and “no handover tool used” (n = 1 [7]). Given the identified evidence gap, the results of those studies reporting any effects of handover tools were reported in this review. In total, 26 studies of the 818 screened records were assessed. In this way, descriptive effects, offering valuable insights into tool utilization were summarized. This broader inclusion facilitated the capture of descriptive trends and patterns in handover tool implementation and effectiveness, enriching our understanding despite the absence of statistically rigorous evidence.

### Study characteristics

The study characteristics are presented in Table 1. Study designs varied, with 13 studies employing a observational study design [8–20], four utilizing qualitative methods [21–24], three involving randomized trials [25–27], and one each for multiphase mixed-method study [28], single-centered controlled study [29], retrospective analysis [30], prospective cohort study [2], mixed methods [28], interventional comparative study [31] and prospective intervention study [32]. In the context of the study settings, 18 of the included studies (69.2%) incorporated (ICUs, while the remaining 8 studies (30.8%) did not have ICUs as part of their settings. Moreover, in relation to the inclusion of polytrauma cases, 5 studies (19.2%) specifically addressed polytrauma patients, whereas the remaining 21 studies (80.8%) focused on different patient populations.

**Table 1:** Study characteristics

Study (year)	Study design	Country	ICU	Polytrauma
Ainsworth et al. (2013)	Observational Pre/post study	USA	Yes	No
Benjamin et al. (2016)	Observational Pre/post study	USA	No	No
Calleja et al. (2020)	Multiphase mixed-method study	Australia	Yes	Yes
Collins et al. (2011)	Qualitative study	USA	Yes	No
Coutsouvelis et al. (2010)	Observational Pre/post study	Australia	Yes	No
Denson et al. (2020)	Single centered controlled study	USA	Yes	No
Ding et al. (2022)	Retrospective analysis	China	Yes	No
Downey et al. (2013)	Randomized controlled trial	USA	No	No
Graan et al. (2016)	Observational Pre/post study	Australia	Yes	No
Haddeland et al. (2022)	Focus group study	Norway	Yes	No
Hoffman et al. (2017)	Observational Pre/post study	USA	Yes	Yes
Kim et al. (2017)	Observational Pre/post study	USA	Yes	No
Li et at. (2022)	Interventional, comparative study	China	No	No
Medlock et al. (2011)	Longitudinal pre/post study	Netherlands	Yes	No
Murphy et al. (2022)	Observational Pre/post study	USA	Yes	No
Parent et al. (2009)	Randomized stepped wedge trial	USA	Yes	No
Patel et al. (2009)	Prospective, observational study	Great Britain	No	No
Prasad et al. (2020)	Observational Pre/post study	USA	Yes	No
Randmaa et al. (2014)	Prospective intervention study	Sweden	Yes	No
Salzwedel et al. (2016)	Prospective, randomized trial	Germany	Yes	No
Schmidt et al. (2021)	Qualitative improvement study	USA	Yes	No
Segall et al. (2016)	Observational Pre/post study	USA	Yes	No
Shahrami et al. (2019)	Observational Pre/post study	Iran	No	Yes
Stahl et al. (2009)	Prospective cohort study	USA	Yes	Yes
Verholen et al. (2021)	Single center, randomized prospective crossover study	USA	No	No
Wayne et al. (2008)	Observational Pre/post study	USA	No	No

The use of the handover tools was tested in various scenarios, which can be subdivided as follows: patients in the ICU without trauma (n = 16), patients in the ICU with trauma (n = 3), patients who were not in the ICU and had no trauma (n = 4), and those who were not in the ICU but had trauma (n = 1).

### Utilized handover tools individual studies

In 12 out of the 26 studies examined, standardized tools were implemented as part of their methodology (see Table 2). The most frequently employed standardized tool was SBAR, which featured in seven studies. One of these studies conducted a direct comparison with the Vicur tool. Additionally, the UW-IPASS, SBARQ, and the Targeted Solution Tool were utilized each in the remaining studies, further exemplifying the diverse range of standardized tools in use.

**Table 2:** Description of Handover tools used in included studies

Author	Tool	Content	Users	Result
Ainsworth et al. (2013)	Door communication board	Basic patient information & major therapy goals	Ward care team	No positive effect
Benjamin et al. (2016)	Targeted Solution Tool	Online application; used for structuring the handover of relevant health information	Physicians	Improvement in communication
Calleja et al. (2020)	SBAR	Standardized form, Information: situation, background, assessment, recommendation	Ward care team	Improvement in communication
Collins et al. (2011)	Customized handover tool	Exchange of documented electronic health records	Physicians, nurses, pharmacists	No positive effect
Coutsouvelis et al. (2010)	Pharmacist initiated pharmaceutical handover (cusromized)	Checklist for medication (dosage, frequency, indication)	Pharmacists and physicians	Reduced therapy errors, positive timely of administration of therapies
Denson et al. (2020)	Customized tool	Checklist	Ward care team	No positive effect
Ding et al. (2022)	SBAR	Standardized form, Information: situation, background, assessment, recommendation	Nursing team	Improvement in information dissemination
Downey et al. (2013)	IPASS	Standardized form; information about illness severity, patient summary, actionlist, situation awareness, synthesis by receiver	Physicians	Improvement in patient therapy satisfaction
Graan et al. (2016)	Customized tool	–	Nursing team	Mitigation of risks
Haddeland et al. (2022)	ISBAR	Standardized form, identify, Information: situation, background, assessment, recommendation	Nursing team and physicians	Improvement in communication
Hoffman et al. (2017)	Customized tool	Visual aid, including risks, comments, and times	Ward care team	Improvement in communication
Kim et al. (2017)	Customized tool	–	–	Improvement in documentation and satisfaction
Li et at. (2022)	SBAR	Standardized form, Information: situation, background, assessment, recommendation	Nursing team	Reduction of referral problems
Medlock et al. (2011)	ICU discharge letter	Letter, all relevant information copied from PDMS	Physicians	Improvement in completeness of patient data & written discharge letters
Murphy et al. (2022)	SBAR	Standardized form, Information: situation, background, assessment, recommendation	Ward care team	Improvement in communication
Parent et al. (2009)	UW-IPASS	Standardized form; information about illness severity, patient summary, actionlist, situation awareness, synthesis by receiver	Physicians	Improvement in communication
Patel et al. (2009)	Customized tool	Electronic tool, –	Physicians	Improvement in information quality
Prasad et al. (2020)	Customized tool	3 step protocol (before, while, after operation); relevant health records	Ward care team	Improvement in communication and psychological safety of ward team
Randmaa et al. (2014)	SBAR	Standardized form, Information: situation, background, assessment, recommendation	Nursing team and physicians	Improvement in communication and reduction of incidents by communication error
Salzwedel et al. (2016)	Customized tool	Checklist and assessment sheet with relevant health records&medical history	Physicians	Improvement in communication
Schmidt et al. (2021)	IPASS	Standardized form; information about illness severity, patient summary, actionlist, situation awareness, synthesis by receiver	Ward care team	Improvement in communication
Segall et al. (2016)	SBAR	Standardized form, Information: situation, background, assessment, recommendation	Ward care team	Improvement of staff satisfaction with handover and reduced clinical workload
Shahrami et al. (2019)	Customized tool	Checklist	Ward care team	Improved quality of information
Stahl et al. (2009)	Customized tool	Checklist with 10 categories	Physicians	Reduction of lost information and communication lapses
Verholen et al. (2021)	SBAR&Vicur	Standardized form, Information: situation, background, assessment, recommendation/Vaccination status	Physicians	No positive effect
Wayne et al. (2008)	Customized tool	n.n	Nursing team	Improvement of information completeness, better perception of accuracy

**Fig. 2:**
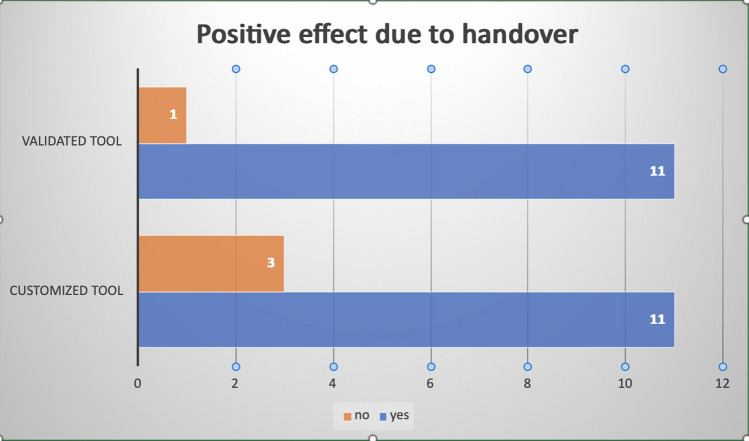
Overview of positive effects reported due to handover tools in hospitals: customized vs. validated tools

In another 12 studies, the focus was assessing the effectiveness of customized handover tools. In the remaining two studies a customized ICU discharge letter and a door communication card were chosen as a tool for handover. Table 2 provides a detailed overview of the tools and strategies employed within the included studies, Fig. 2 gives an overview of the positive effects reported.

### Treatment improvement

Two types of handover tools were identified: custom-designed tools and well-established validated tools. Among the studies that employed custom-designed tools, three did not identify any positive effects [13, 27, 29]. These outcomes included enhancements in information quality and quantity, improved communication among healthcare staff, as well as increased psychological safety and perceptions of accuracy and completeness. Additionally, two studies explicitly noted a reduction in risks and a decrease in treatment errors, see Table 2 [15, 16].

One study, which compared different standardized tools while also assessing their overall impact, did not find any positive effect [27]. Among the studies that employed a well-established standardized tool, twelve studies documented beneficial outcomes [2, 9, 14, 17, 21, ,25–28, 30–32]. These positive results were characterized by improvements in communication and documentation, heightened satisfaction among medical professionals regarding handovers, and enhancements in managing challenging cases and referral issues. Additionally, these studies observed a reduction in incidents of lost information and communication lapses, see Table 2.

## Discussion

Our systematic review aimed to assess the utility of handover tools in healthcare settings, a topic of critical importance in improving patient care. We performed a systematic search, conform to the Cochrane standard, that yielded a substantial number of records, demonstrating the significance and relevance of the subject matter. However, during the review process, it became evident that there were no randomized controlled trials or systematic reviews analyzing the effect of the use of handover tools on the treatment of polytrauma patients. Further, a significant number of studies was excluded because they did not meet the specific definition of "multiple trauma" and instead focused on broader or different clinical conditions, a problem that has been repeatedly demonstrated in relation to the treatment of polytrauma patients, as there is a lack of standardised and valid definitions for this patient group. Without the appropriate studies and a standardised definition, no recommendations for action can be made for individual patient groups. Given the identified gap in the available evidence, we reported the findings related to the general impact of handover tools in healthcare settings as presented by the studies reviewed. These studies exhibited a remarkable diversity in study design, context, and outcomes. This diversity made it challenging to conduct a conventional meta-analysis or extract quantitative data for predefined outcomes. Instead, our review offers insights into the unique challenges encountered when attempting to consolidate evidence in an area where studies exhibit significant heterogeneity and variability. The limited number of eligible studies is a notable finding and indicates a gap in the existing literature on this topic.

Among the studies that explored customized handover tools, it is evident that the majority reported positive outcomes. Specifically, eleven out of twelve studies employing custom-designed tools documented beneficial effects. These encompassed improved information quality and quantity, enhanced communication among healthcare staff, and an increased sense of psychological safety, accuracy, and completeness. Notably, two studies reported a reduction in risks and treatment errors. These findings underscore the potential of tailored handover tools to enhance various facets of healthcare delivery and interprofessional communication. In parallel, the use of well-established standardized tools also yielded promising results. Eleven out of twelve studies reported positive outcomes. These included enhancements in communication and documentation, heightened satisfaction among medical professionals regarding handovers, and improved management of complex cases and referral issues. Additionally, these studies noted a reduction in incidents of lost information and communication lapses. It is worth noting that one study, which compared different standardized tools while also assessing their overall impact, did not find any positive effect.

The comparative findings indicate that both customized and standardized tools have their strengths, offering flexibility and consistency, respectively. Selecting the appropriate handover tool depends on the specific needs of healthcare organizations and the clinical contexts they serve. Future research could further explore the comparative effectiveness of these tools and consider how a combination of customized and standardized approaches can optimize healthcare handovers.

It is essential to acknowledge the limitations of this review. The limited number of eligible studies and the variability in study designs make it challenging to draw definitive conclusions about the utility of handover tools. Additionally, publication bias may have influenced the availability of relevant studies. Despite these limitations, this review highlights the importance of further research in this area. Future studies should aim to standardize methodologies and outcomes to facilitate more robust comparisons across different handover tools. Moreover, exploring the factors influencing the effectiveness of custom-designed tools could provide valuable insights for healthcare practitioners and policymakers. So, while this review may not provide the conventional statistical evidence typically associated with meta-analysis, it serves a valuable purpose. It highlights the complexities of the research landscape and underscores the need for a more tailored approach when examining topics with diverse study designs and outcomes.

## Conclusion

Heterogeneity of the studies and limited number of eligible trials present challenges for conducting a traditional systematic review. Still this review sheds light on the complexities of assessing handover tools’ utility, especially in diverse clinical settings. It highlights the need for more standardized methodologies and further investigation into the effectiveness of custom-designed tools. It emphasizes the importance of understanding the role of handover tools in healthcare. While some studies suggest positive outcomes, further research is necessary to elucidate the design and implementation of these tools to enhance care and support healthcare professionals in their roles. A first step could involve expert consensus through a Delphi process to develop an outline for an effective and standardized handover tool.

## Data Availability

Additional study data and search strings are available from the corresponding author.

## Appendix 1

Search Strategy.

## Abbreviations

ICU: Intensive care unit
ISS: Injury severity score
RCT: Randomized controlled trial
RoB: Risk of bias
DGAI: German Society for Anaesthesiology and Intensive Care Medicine (Deutsche Gesellschaft für Anästhesiologie und Intensivmedizin)
DIVI: German Interdisciplinary Association of Critical Care and Emergency Medicine (Deutsche Interdisziplinäre Vereinigung für Intensiv- und Notfallmedizin)
